# Neuropsychiatric Outcomes in Intensive Care Unit Survivors

**DOI:** 10.7759/cureus.40693

**Published:** 2023-06-20

**Authors:** Shivam Joshi, Ravi Prakash, Zia Arshad, Monica Kohli, Gyan Prakash Singh, Neelam Chauhan

**Affiliations:** 1 Department of Anesthesiology and Critical Care, King George's Medical University, Lucknow, IND

**Keywords:** anxiety, depression, intensive care unit, health related quality of life (hrqol), neuropsychiatry

## Abstract

Background: Over the last two decades, there has been phenomenal advancement in critical care medicine and patient management. Many patients recover from life-threatening illnesses that they might not have survived a decade ago. Despite a decrease in mortality, these survivors endure long-lasting sequelae like physical, mental, and emotional symptoms.

Methods: Patients after intensive care unit (ICU) discharge were assessed in a follow-up outpatient department (OPD) clinic for anxiety, stress, and depression. Patients were asked to fill out the questionnaires Depression, Anxiety and Stress Scale-21 (DASS-21) and Short Form-36 (SF-36) for assessment of health-related quality of life (HRQOL) at 4th, 6th, and 8th months after discharge. ICU data were recorded, including patients’ demographics, severity of illness and length of stay, and duration of mechanical ventilation. Patients who failed to follow-up in OPD on designated dates were assessed telephonically.

Results: Depression showed a positive, strong, and moderate correlation between length of stay and mechanical ventilation duration. A positive correlation was found between stress and length of stay and duration of mechanical ventilation. A positive strong correlation was found between anxiety and length of ICU stay, and a moderate positive correlation was found between anxiety and duration of mechanical ventilation. A weak correlation was found between age and neuropsychiatric outcomes.

Conclusion: The severity of depression, anxiety, and stress was significantly higher at four months compared to six months. Severity decreased with time. Prolonged ICU stay increased levels of anxiety, depression, and stress. HRQOL improved from four to six months.

## Introduction

Over the last two decades, there have been phenomenal advancements in critical care medicine and patient management in the ICU. In fact, many patients recover from life-threatening illnesses that they might not have survived a decade ago [1]. However, very little attention has been paid to long-term sequelae in ICU survivors. Those who survive are frequently unable to regain their health and report a decline in quality of life [2].

Additionally, the psychological repercussions of critical diseases are being investigated with increased rigor [3]. Despite a decrease in mortality, survivors of severe illness typically endure long-lasting sequelae [4]. The pathophysiology of the disease, organ dysfunction that develops during hospitalization, or any dysfunction that develops during the ICU stay and/or during prolonged intensive care support for failing organ(s) can induce these consequences [5]. The spectrum of problems may be diverse and persistent, including reduced pulmonary function, neuromuscular weakness, neuropsychiatric complications, depression, and posttraumatic stress disorder [6,7]. Depression, anxiety, and stress are psychological effects of intensive care. Psychological issues negatively affect perceptions of future health status and HRQOL [8]. Peri-ICU and post-ICU therapies have the potential to enhance neuropsychiatric outcomes for ICU survivors [9,10]. Little data exists on the long-term neuropsychological consequences of sepsis. A total of 80% of acute respiratory distress syndrome (ARDS) survivors in one cohort were found to have impaired memory, attention, concentration, or processing speed one year after hospital discharge [11], and nearly 25% were found to have mild cognitive impairment six years after discharge [12]. The aims of the study were to estimate the incidence of neuropsychiatric outcomes; to evaluate the symptoms of anxiety, depression, and stress; and to examine the HRQOL in ICU patients.

## Materials and methods

**Figure 1:**
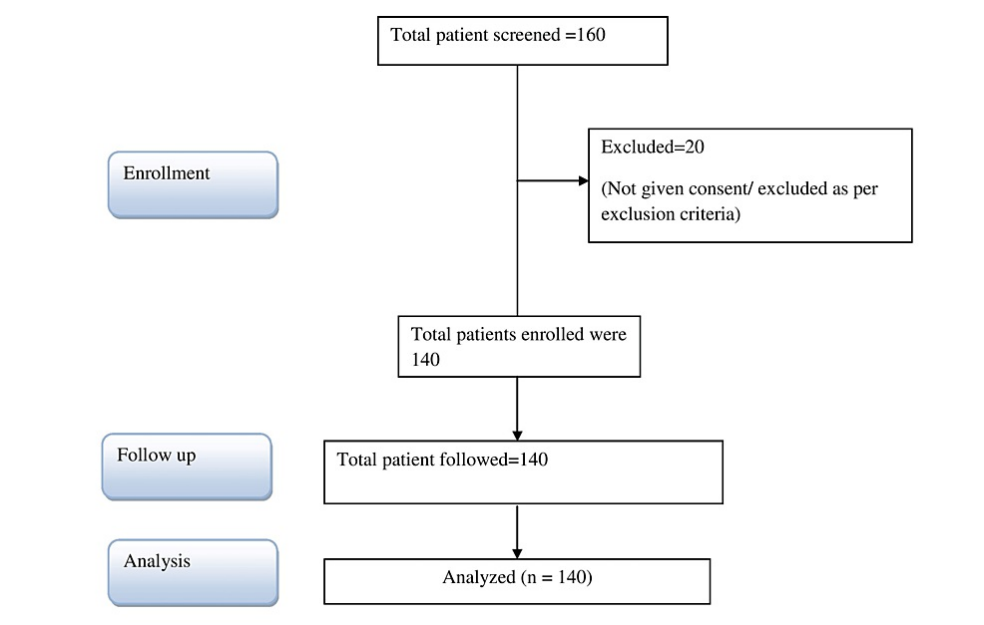
Flowchart of participants.

This prospective cohort study was conducted at follow-up clinics (FUCs) in tertiary-level ICUs over a period of one year and one month (December 2021 to January 2023). FUCs deal with patients who were discharged to home directly from the ICU. After obtaining ethical approval from the Institutional Ethics Committee (approval number: VI-PGTSC-IIA/P45), patients were enrolled in the first five months of the study and then followed up for eight months (Figure 1). Written and informed consent was obtained from attendants/patients for participation in the study, and for use of patient data for research and educational purposes. The guidelines laid down in the Declaration of Helsinki (1964) were followed in the procedure. Adult patients of either sex admitted in the ICU for more than 48 hours were screened and enrolled after considering exclusion criteria. Unwilling patients, dropout patients, patients already having any neuropsychiatric illness before admission, patients having any mental trauma during the study period that affected their psychological status, patients with an inability to communicate, and patients with all head injuries were excluded from the study.

Patients after ICU discharge were assessed in a follow-up in OPD for anxiety, stress, and depression. Patients were asked to fill out the questionnaires - Depression, Anxiety and Stress Scale-21 (DASS-21) and Short Form-36 (SF-36) for assessment of HRQOL at fourth, sixth, and eighth months after discharge. Detailed ICU data were recorded, including patients’ demographics, severity of illness at ICU admission, length of ICU stay, and duration of mechanical ventilation. Patients who did not follow-up in OPD on designated dates were reminded telephonically to follow-up in the next seven days, or they were assessed telephonically itself. Patients discharged four months back were included in this study and were asked for follow-up in OPD. Patients with two consecutive follow-ups in fourth and sixth months were included in this study. Statistical analysis was conducted using SPSS statistical analysis software version 15.0 (Armonk, NY: IBM Corp.). The values were represented in number (%) and mean±SD.

## Results

A total of 160 patients were screened, and 20 patients were excluded according to defined exclusion criteria. A total of 140 patients were enrolled in the study and followed up for six months. Out of 140, only 10 were followed up from six months to eight months for various reasons. Thus, eight-month data were not included in the study. Most of the patients were aged between 28 and 37 years (58 {41.43%}) and 18 and 27 years (40 {28.57%}). A total of 17.14% were in the age group of 38-47 years. Only three patients were in the age group of 58-65 years. Most of the patients were females (81 {57.86%}) and the rest were males (59 {42.14%}) (Table 1).

**Table 1:** Age and gender-wise distribution of enrolled patients who had ICU stays for more than 48 hours.

Variables		Number	Percentage
Age in years	18-27	40	28.57%
	28-37	58	41.43%
	38-47	24	17.14%
	48-57	15	10.71%
	58-65	3	2.14%
	Grand total	140	100.00%
Gender	Female	81	57.86%
	Male	59	42.14%
	Grand total	140	100.00%

**Figure 2:**
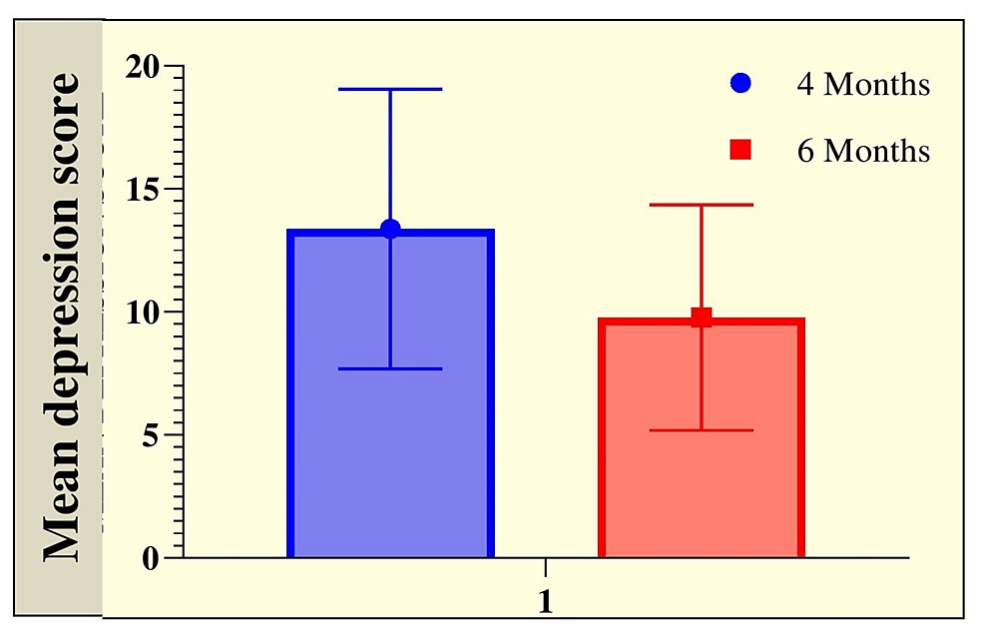
Graphical representation of the mean severity of depression in enrolled patients at follow-up of four and six months.

After four months, mild depression was found in 34.29% of patients and moderate depression was found in 14.29% of patients based on DASS-21 scale. The majority of patients were normal (48.57%). At six months, 64.29% of patients were normal, 26.43% of patients had mild depression, and 6.43% of patients had moderate depression. At the same time, at four months, four patients had severe depression, and this number remained constant even at six months. Mean depression was higher at four months (13.37±5.67) than at six months (9.78±4.58). Statistically, a significant difference was observed in the severity of depression (Table 2 and Figure 2).

**Table 2:** Severity of depression in enrolled patients at follow-up of four and six months.

Depression	4 months (n=140)		6 months (n=140)		p-Value
	n	%	n	%	
Normal (0-9)	68	48.57%	90	64.29%	0.0342
Mild (10-13)	48	34.29%	37	26.43%	
Moderate (14-20)	20	14.29%	9	6.43%	
Severe (21-27)	4	2.86%	4	2.86%	
Extremely severe (≥28)	0	0.00%	0	0.00%	
Mean±SD	13.37±5.67		9.78±4.58		t=5.828, p<0.0001

**Figure 3:**
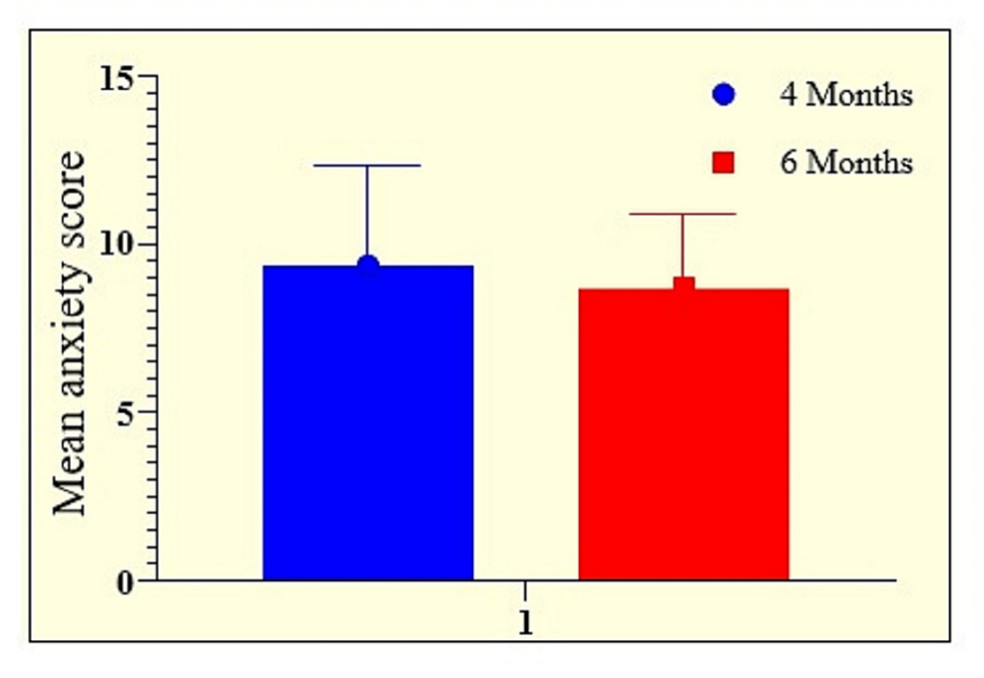
Graphical representation of the mean severity of anxiety in enrolled patients at follow-ups of four and six months.

More than half of the patients experienced mild anxiety (51.43%), 21.43% of patients experienced moderate anxiety, and 4.29% of patients experienced severe anxiety. At six months, mild anxiety was found in 46.43% of patients, moderate anxiety was found in 14.29% of patients, and severe anxiety was found in only three patients (2.14%). Similarly, 4.29% of patients showed severe anxiety at four months, which had decreased by six months. Mean anxiety was higher at four months (9.38±2.94) than six months (8.69±2.19). Statistically, a significant difference was observed in the severity of anxiety (Table 3 and Figure 3).

**Table 3:** Severity of anxiety in enrolled patients at follow-up of four and six months.

Anxiety	4 months (n=140)		6 months (n=140)		p-Value
	n	%	n	%	
Normal (0-7)	32	22.86%	52	37.14%	0.0436
Mild (8-9)	72	51.43%	65	46.43%	
Moderate (10-14)	30	21.43%	20	14.29%	
Severe (15-19)	6	4.29%	3	2.14%	
Extremely severe (≥20)	0	0.00%	0	0.00%	
Mean±SD	9.38±2.94		8.69±2.19		t=2.227, p=0.0267

**Figure 4:**
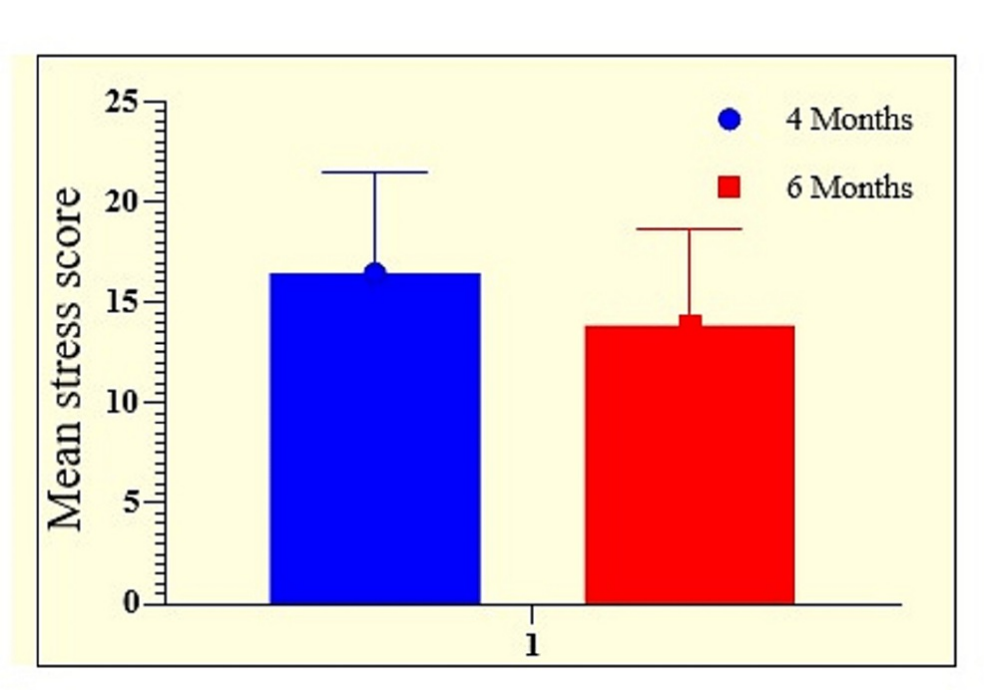
Graphical representation of the mean severity of stress in enrolled patients at follow-ups of four and six months.

At four months, half of the patients (50%) reported mild stress, 20.7% of patients reported moderate stress, and 4.29% of patients reported severe stress. At six months, 47.86% of patients experienced mild stress, 12.86% of patient’s experienced moderate stress, and only 1.43% of patients experienced severe stress. Mean stress was higher at four months (16.47±5.04) than six months (13.89±4.81). Statistically, a significant difference was observed in the severity of stress at four months and six months (Table 4 and Figure 4).

**Table 4:** Severity of stress in enrolled patients sat follow-up of four and six months.

Stress	4 months (n=140)		6 months (n=140)		p-Value
	n	%	n	%	
Normal (0-14)	35	25.00%	53	37.86%	0.0398
Mild (15-18)	70	50.00%	67	47.86%	
Moderate (19-25)	29	20.71%	18	12.86%	
Severe (26-33)	6	4.29%	2	1.43%	
Extremely severe (≥34)	0	0.00%	0	0.00%	
Mean±SD	16.47±5.04		13.89±4.81		t=4.382, p<0.0001

The mean length of ICU stays was recorded as 6.01±4.43 days. The majority of patients had <five days of ICU stay (63.57%), followed by 5-10 days of ICU stay (63.57%). The majority of the patients who had <five days of ICU stay had anxiety (24%) followed by stress (36%) and depression (21%). Patients with more than five days of ICU stay had more anxiety, stress, and depression (Table 5).

**Table 5:** Association between length of ICU stays of enrolled patients and neuropsychiatric outcomes.

Length of ICU stay	Anxiety	Stress	Depression
<5 days	24%	36%	21%
5-10 days	40%	40%	32%
>10 days	62%	42%	56%

**Figure 5:**
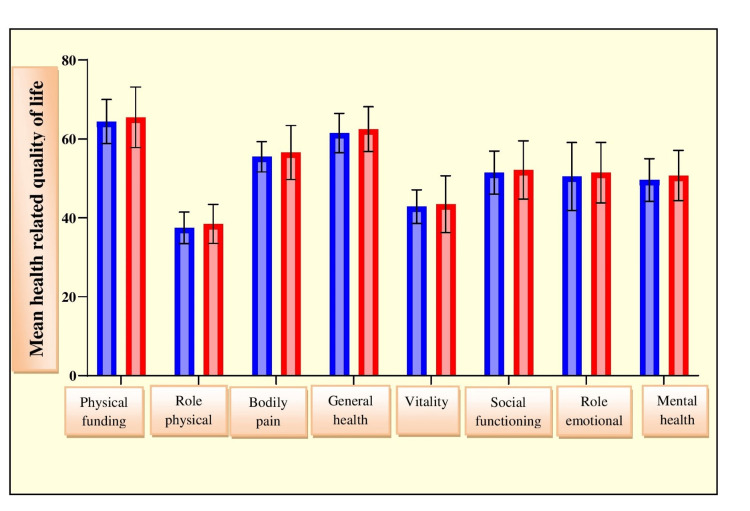
Graphical representation of health-related quality of life of enrolled patients at follow-ups of four and six months.

The mean duration of ventilation was recorded as 2.25±3.68 days. A total of 97.14% of patients had 0-5 days of duration of mechanical ventilation, and 2.14% of patients had 5-10 days of duration of mechanical ventilation. At four months, physical functioning was 64.41±5.58, body pain was 55.48±3.84, general health was 61.48±4.98, mental health was 49.58±5.41, and so on. At six months, improvement was observed in patients. Physical functioning was 65.48±7.68, body pain was 56.58±6.84, general health was 62.48±5.66, mental health was 50.72±6.39, and so on. Statistically, a nonsignificant difference was observed in HRQOL (Table 6 and Figure 5).

**Table 6:** Health-related quality of life of enrolled patients at follow-ups of four and six months.

Health-related quality of life	SF-36 questionnaire				p-Value
	At 4 months		At 6 months		
	Mean	SD	Mean	SD	
Physical functioning	64.41	5.58	65.48	7.68	t=1.33, p=0.18
Role physical	37.48	3.98	38.48	4.94	t=1.86, p=0.06
Body pain	55.48	3.84	56.58	6.84	t=1.65, p=0.09
General health	61.48	4.98	62.48	5.66	t=1.56, p=0.11
Vitality	42.84	4.24	43.48	7.17	t=0.90, p=0.36
Social functioning	51.48	5.44	52.14	7.38	t=0.85, p=0.39
Role emotional	50.47	8.61	51.48	7.67	t=1.03, p=0.30
Mental health	49.58	5.41	50.72	6.39	t=1.61, p=0.10

**Figure 6:**
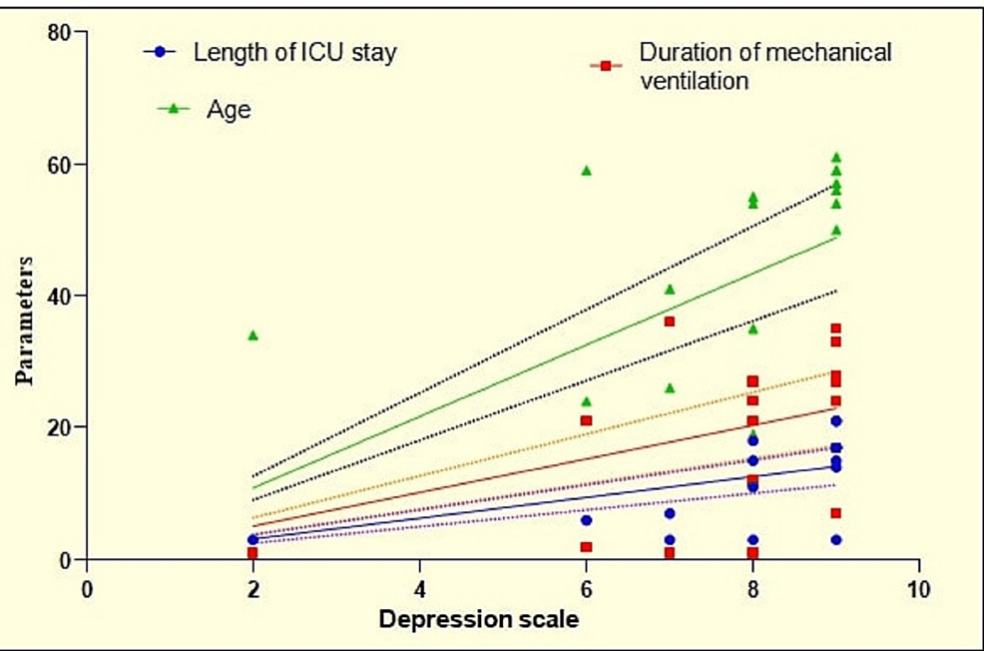
Graphical representation of correlation analysis of depression with other parameters.

The correlation analysis showed a significantly positive strong correlation between depression and length of ICU stay (p=0.004, r=0.6283). A significantly positive moderate correlation was observed between depression and duration of mechanical ventilation (p=0.0293, r=0.4998). A weak correlation was observed with age, but it was not significant (Table 7 and Figure 6). 

**Table 7:** Correlation analysis of the depression, anxiety, and stress scale with other parameters.

Variables		Spearman r	Correlation	95% confidence interval	p-Value
Depression scale	Length of ICU stay (days)	0.6283	Strong	0.2300 to 0.8463	0.004
	Duration of mechanical ventilation (hours)	0.4998	Moderate	0.04450 to 0.7832	0.0293
	Age (years)	0.3802	Weak	-0.1038 to 0.7186	0.1083
Stress scale	Length of ICU stay (days)	0.6746	Strong	0.1791 to 0.8971	0.0138
	Duration of mechanical ventilation (hours)	0.5597	Moderate	-0.005686 to 0.8539	0.0494
	Age (years)	0.3884	Weak	-0.2244 to 0.7810	0.1883
Anxiety scale	Length of ICU stay (days)	0.6809	Strong	0.1569 to 0.9058	0.0177
	Duration of mechanical ventilation (hours)	0.5117	Moderate	0.03898 to 0.8819	0.0378
	Age (years)	0.3317	Weak	-0.07995 to 0.8525	0.1781

**Figure 7:**
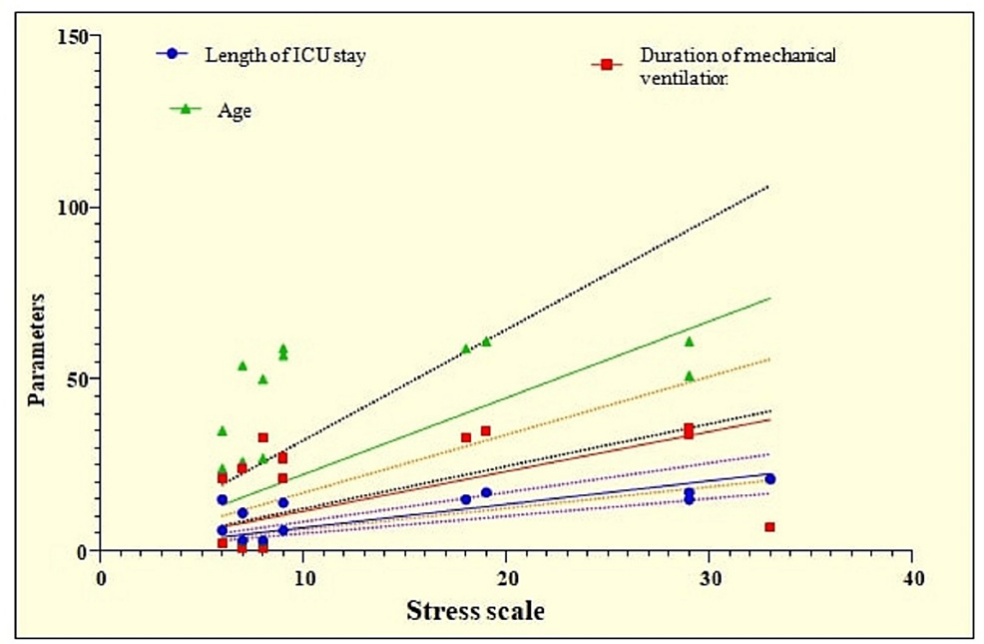
Graphical representation of correlation analysis of stress with other parameters.

The correlation analysis showed a significantly positive correlation between stress and length of ICU stay (p=0.0138, r=0.6746). A significantly positive moderate correlation was observed between stress and duration of mechanical ventilation (p=0.0494, r=0.5597). A weak correlation of stress, anxiety, depression was observed with age but it was not significant (Table 7 and Figure 7).

**Figure 8:**
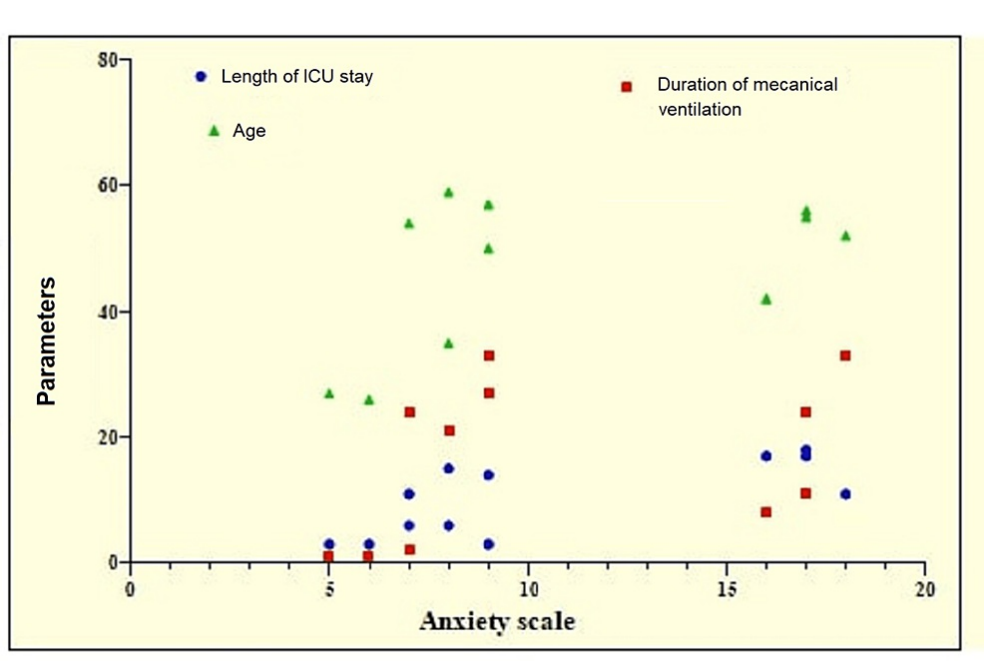
Graphical representation of correlation analysis of anxiety with other parameters.

The correlation analysis showed a significantly positive strong correlation between anxiety and length of ICU stay (p=0.0177, r=0.6809). A significantly positive moderate correlation was observed between anxiety and duration of mechanical ventilation (p=0.0378, r=0.5117). A weak correlation of stress, anxiety, and depression was observed with age, but it was not significant (Table 7 and Figure 8).

## Discussion

A total of 50% of patients admitted to ICU for a minimum of one week are reported to have physical impairments. A total of 20-40% of ICU survivors develop permanent cognitive impairments [13]. Delirium is a well-known cognitive consequence of ICU admission, affecting up to 75% of ICU patients and occurring more frequently in mechanically ventilated patients [14]. After discharge, up to one-third of ICU survivors may develop a variety of psychological dysfunctions [15]. Post-ICU sadness affects around 30% of ICU survivors and is associated with higher hospital admissions and trips to the emergency department [16]. Anxiety has an estimated 70% prevalence of anxiety among ICU survivors. Patients with post-ICU anxiety frequently suffer from post-ICU depression or PTSD [17]. The prevalence of PTSD following ICU care ranges from 10% to 50%. Numerous individuals with post-ICU anxiety continue to experience anxiety symptoms one year after being discharged [18].

The DASS is a self-report scale with 21 items [19]. It was created to measure the intensity of depression’s and anxiety’s primary symptoms. A third element called “stress” arose during the iterative development phase. Psychometric features of the DASS and DASS-21 have been proven in clinical and nonclinical groups [19-22] as well as in younger adults with pain [23]. The DASS-21 has also been validated in a group of primary care patients aged 65 years and older [24].

The SF-36 is a health status profile that was originally designed to measure patients’ health status and outcomes. The 36 questions are meant to reflect eight domains of health-physical functioning, physical role, pain, general health, vitality, social function, emotional role, and mental health for scoring purposes [25]. Analysis and interpretation of the resulting linear scales presume that, on average, item scores have a linear relationship with the underlying health concept being measured. The research conducted so far supports this assumption for SF-36 items [26]. To improve the display and comprehension of a health profile, all scales are evaluated favorably. The SF-36 scoring manual, which is accessible from the senior author, contains guidelines for scoring items and scales [27]. A modest but substantial body of evidence has shown persistent neuropsychological damage in critical illness survivors [28]. Acute encephalopathy has been observed in up to 70% of sepsis patients [29,30], and generalized cognitive deficits have been described in individuals with toxic shock syndrome [31]. In a study by Heesakkers et al., 302 patients who survived ICU treatment were enrolled, with the majority aged 16 years or older [27]. According to the study by Osman et al. in 2012, the mean age of patients was 43±13 years [32]. Additionally, they noticed male dominance (n=77).

Another study found that approximately 25% of ARDS survivors had mild cognitive impairment six years after their ICU stay [33,34]. However, no prospective data describes cognitive impairment in the overall ICU population. In the current study, the majority of patients were between the ages of 28 and 37 years (58 {41.43%}), followed by those between 18 and 27 years (40 {28.57%}). A total of 17.14% were between the ages of 38 and 47 years. Only three patients between the ages of 58 and 65 were observed. The majority of patients were women (81 {57.86%}), followed by men (59 {42.14%}). The mean age was determined to be 61.2±9.3 years. Another study by Dijkstra-Kersten et al. showed that the mean age of the patients was 59±16 years and that 65% of the patients were male [35]. There was a weak and insignificant correlation between age and neuropsychiatric outcomes. In this study, the mean length of ICU stays was recorded as 6.01±4.43 days, with the majority of patients (63.57%) having stays of <five days, followed by stays of 5-10 days.

According to Dijkstra-Kersten et al., the average length of stay in an ICU was 5.3 days [35]. A large percentage of patients who spent less than five days in the ICU showed anxiety (24%), followed by stress (36%) and depression (21%). In our study, the mean duration of mechanical ventilation was 2.25±3.68 days. A total of 97.14% of patients required mechanical ventilation for 0-5 days, whereas 2.14% required mechanical ventilation for 5-10 days. Most studies on post-ICU follow-up interventions have focused on mechanically ventilated ICU survivors or survivors with a lengthy ICU stay (34 days). After four months, mild depression was found in 34.29% of patients, and moderate depression was found in 14.29%. The majority of patients were normal (48.57%). At six months, 64.29% of patients were normal, 26.43% of patients had mild depression, and 6.43% of patients had moderate depression. Four patients developed significant depression at four months, which persisted at six months. The mean level of depression was greater after four months (13.37±5.67) than at six months (9.78±4.52). The higher incidence of depression at four months can be explained by patients’ recovery from poor health and high expenditure in ICU. As time passes, these patients returned to normalcy and became more involved in their daily life, leading to decreased incidence at six months. In the present study, compared to Heesakkers et al.’s study, a significantly positive strong correlation between depression and ICU length of stay (p=0.004, r=0.6283) and a significantly positive moderate correlation between depression and duration of mechanical ventilation (p=0.0293, r=0.4995) were observed [27]. Those authors evaluated outcomes using Hospital Anxiety and Depression Scale (HADS) and found that one year after ICU treatment for COVID-19, 17.9% of survivors experienced anxiety symptoms, and 18.3% reported depression. Moreover, 9.8% of survivors exhibited PTSD symptoms.

More than half of the patients experienced mild anxiety (51.43%), 21.43% experienced moderate anxiety, and 4.29% experienced severe anxiety. The rest of the patients were normal. At six months, mild anxiety was found in 46.43% of patients, moderate anxiety was found in 14.29% of patients, and severe anxiety was found in only three patients. Similarly, 4.29% of patients showed severe anxiety at four months, which had decreased by six months. The average level of anxiety was greater at four months (9.38±2.94) compared to six months (8.69±2.20). Correlation analysis showed a significantly positive strong correlation between anxiety and length of ICU stay (p=0.0177, r=0.6809). A significantly positive moderate correlation was observed between anxiety and duration of mechanical ventilation (p=0.0378, r=0.5117).

In the current study, at four months, half of the patient population (50%) reported mild stress, 20.7% reported moderate stress, and 4.29% reported severe stress. The rest of the patients were normal. At six months, 47.86% patients experienced mild stress, 12.86% patients experienced moderate stress, and only 1.43% patients experienced severe stress. The mean stress level was greater at four months (16.475.04) than at six months (13.894.81). Correlation analysis showed a significantly positive correlation between stress and length of ICU stay (p=0.0138, r=0.6744). The correlation between stress and duration of mechanical ventilation was found to be moderately significant (p=0.0494, r=0.5597). Physical functioning was 64.41±5.58, bodily pain was 55.48±3.84, general health was 61.48±4.98, and mental health was 49.58±5.41 at four months. At six months, patients showed signs of improvement. Physical functioning improved (65.48±7.68), as did body pain (56.58±6.84), general health (62.48±5.66), mental health (50.72±6.39), vitality, social functioning, physical role, emotional role, and social functioning, although the difference among these was not statistically significant.

Moreover, Davydow et al. repeatedly showed that nearly three-quarters of the patients in their study with significant PTSD symptoms three months after being discharged from the ICU reported that their symptoms were related to their memories of in-ICU events [36]. This proportion remained above 40%, nine months later. According to Sukantarat et al., 22-31% of intensive care survivors exhibited symptoms of anxiety and depression nine months later [37]. Approximately one-fourth of patients who survive a three-day ICU stay will exhibit persistent psychiatric issues for at least nine months after discharge. Davydow et al. observed PTSD related to sedatives, notably benzodiazepine, because its administration may represent physicians’ treatment of patients’ in-ICU anxiety [36]. This may be a risk factor for PTSD or a precursor symptom. Survivors of ICUs may be offered customized follow-up interventions depending on their risk profile and personal preference to individualize therapy and optimize resource use [10,23]. FUCs can play a vital role in diagnosing and managing medical problems of ICU survivors. Early diagnosis of neuropsychiatric illness in these patients can allow early counseling and treatment, preventing long-term complications. There are many other problems with patients that we regularly observe in our follow-up clinic (FUC), and they need a separate management approach. The severity of depression, anxiety, and stress was significantly higher at four months compared to six months. Severity decreased with time. Prolonged ICU stays increased the severity of anxiety, depression, and stress. HRQOL was improved from four to six months. However, the difference was nonsignificant. Correlation analysis showed a positive strong correlation between depression and length of ICU stay. A significantly positive moderate correlation was observed between depression and duration of mechanical ventilation. Correlation analysis showed a significantly positive correlation between stress and length of ICU stay. A significantly positive moderate correlation was observed between stress and duration of mechanical ventilation. Correlation analysis showed a positive strong correlation between anxiety and length of ICU stay.

A significantly positive moderate correlation was observed between anxiety and duration of mechanical ventilation. A weak and insignificant correlation was found between age and neuropsychiatric outcomes. Follow-up OPD for ICU survivors may also help in reducing neuropsychiatric illnesses like anxiety, stress, and depression. Regarding the limitations of this study, it lacked randomization and blinding, which may have contributed to a degree of bias in case selection. Results were limited to a single tertiary care hospital and may not be generalized to all settings. To establish a relationship between neuropsychiatric outcomes in ICU survivors, large prospective studies are required.

## Conclusions

In this prospective cohort study, it was observed that there was an increase in levels of anxiety, stress, and depression. This increase was directly proportional to the length of ICU stay and duration of mechanical ventilation. However, the severity of neuropsychiatric symptoms decreased as time elapsed. Regular follow-ups in ICU clinics may help in the early detection and prompt treatment of such problems and help in improving quality of life.
